# Mesenchymal stem cells in rabbit meniscus and bone marrow exhibit a similar feature but a heterogeneous multi-differentiation potential: superiority of meniscus as a cell source for meniscus repair

**DOI:** 10.1186/s12891-015-0511-8

**Published:** 2015-03-21

**Authors:** Zhe Ding, He Huang

**Affiliations:** Department of Ophthalmology, The 3rd Affiliated Hospital of Nanjing University of Traditional Chinese Medicine, 1 Jinling Road, Nanjing, Jiangsu 210001 China; Department of Orthopaedic Surgery, Nanjing First Hospital, Nanjing Medical University, 68 Changle Road, Nanjing, Jiangsu 210006 China

**Keywords:** Meniscus-derived mesenchymal stem cells, Bone-marrow derived mesenchymal stem cells, Chondrogenesis, Meniscal regeneration

## Abstract

**Background:**

The restoration of damaged meniscus has always been a challenge due to its limited healing capacity. Recently, bone marrow-derived mesenchymal stem cells (BMSCs) provide a promising alternative to repair meniscal defects. However, BMSCs are not ideal chondroprogenitor cells for meniscus repair because they have a high propensity for cartilage hypertrophy and bone formation. Our hypothesis is that mesenchymal stem cells (MSCs) reside in meniscus maintain specific traits distinct from others which may be more conducive to meniscus regeneration.

**Methods:**

MSCs were isolated from bone marrow and menisci of the rabbits. The similarities and differences between BMSCs and MMSCs were investigated *in vitro* by a cell culture model, *ex vivo* by a rabbit meniscus defect model and *in vivo* by a nude rat implantation model using histochemistry, immunocytochemistry, qRT-PCR and western blotting.

**Results:**

Our data showed that two types of MSCs have universal stem cell characteristics including clonogenicity, multi-potency and self-renewal capacity. They both express stem cell markers including SSEA-4, Nanog, nucleostemin, strol-1, CD44 and CD90.

However, MMSCs differed from BMSCs. MMSC colonies were much smaller and grew more slowly than BMSC colonies. Moreover, fewer MMSCs expressed CD34 than BMSCs. Finally, MMSCs always appeared a pronounced tendency to chondrogenic differentiation while BMSCs exhibited significantly greater osteogenic potential, whatever *in vitro* and *in vivo*.

**Conclusions:**

This study shows the similarities and differences between MMSCs and BMSCs for the first time. MMSCs are a promising source of mesenchymal stem cells in repairing meniscus defect.

**Electronic supplementary material:**

The online version of this article (doi:10.1186/s12891-015-0511-8) contains supplementary material, which is available to authorized users.

## Background

The knee meniscus which consists of two semilunar, wedge-shaped pieces fixed between femoral condyle and tibial plateau plays a crucial role in the function of knee joint. Injury or loss of meniscus can lead to osteoarthritis and irreversible joint damage [1]. Several studies showed that even partial meniscectomy can significantly increase the incidence of osteoarthritis [2]. At present, there is an ever increasing emphasis on meniscus preservation through surgical repair [3].

Unfortunately, because the meniscus is not a homogeneous tissue, capability of self-healing in vascular or avascular part is much different. While tears that occur in the outer periphery of the tissue can regenerate due to the high degree of vasculature there, damage to the inner non-vascularized portion of the tissue is difficult to heal on its own [4]. Even repaired with different kinds of new techniques, healing response in avascular zone of meniscus was still absent [5]. The successful surgical restoration of the damaged meniscus still remains an ongoing challenge.

Recently, meniscal regeneration using mesenchymal stem cells (MSCs)-based tissue engineering techniques have been attempted, which provide a promising alternative for repair of meniscal defects [6]. In both clinical and experimental perspectives, MSCs have received the most attention due to their multi-differentiation potential. Although ever-increasing amount of literature has shown that different kinds of MSCs, including bone marrow-derived MSCs (BMSCs), synovium-derived MSCs (SMSCs) and adipose-derived MSCs (AMSCs) may all prove beneficial for meniscus repair due to their evident chondrogenic capacity [7-10], none of them has received a general consensus in literature. Indeed, the search for effective therapies based on tissue engineering approaches is still a debated and investigational issue. The key is to find a kind of novel MSCs which will satisfactorily integrate with the host, and which will allow the long-term preservation of cell viability.

Based on the previous studies, we hypothesize that MSCs may reside in meniscus, and those meniscus-derived MSCs (MMSCs) should maintain specific traits distinct from other MSCs which may improve meniscus regeneration. However, knowledge of MMSCs still remains limited, and no studies to date have evaluated their effect on the regeneration of meniscus *in vivo* through compared to BMSCs. To test our hypothesis, we (1) isolated the MMSCs and BMSCs from the same rabbit and demonstrated that MMSCs possessed features typical of stem cells, (2) evaluated their respective multi-differentiative potential *in vitro*, (3) detected their regenerative capacity when seeded in the defect of wounded meniscus within the rabbit model, and (4) investigated their effect of multi differentiation after their implantation into the back of nude rats.

## Methods

### Isolation of meniscus-derived mesenchymal stem cells (MMSCs) and bone marrow stem cells (BMSCs)

Ten female New Zealand white rabbits (8–10 week-old, 3.0 - 4.0 kg) were used in all experiments. The protocol for use of the animals was approved by the IACUC of the Nanjing Medical University. The rabbits were fully sedated by intra-muscular injection of Ketamine (10 mg/kg) and Xylazine (3 mg/kg) and were then sacrificed using pentobarbital (120 mg/kg). After sacrifice, rabbit medial and lateral menisci were dissected from bilateral knee joints. For isolation of MMSCs, the parameniscus tissues were removed, and the whole meniscus was then weighed and minced into small pieces (1 mm × 1 mm × 1 mm). Each 100 mg tissue sample was digested with 3 mg collagenase type I (Worthington Biochemical Corporation, Lakewood, NJ) and 4 mg dispase (StemCell technologies Inc., Vancouver, BC, Canada) in 1 ml phosphate buffered saline (PBS) at 37 °C for 1 hr. The suspensions were centrifuged at 1,500 g for 15 min, and the supernatant was discarded. The remaining cell pellet was re-suspended in growth medium consisting of Dulbecco’s modified Eagle’s medium (DMEM; Lonza, Walkersville, MD) supplemented with 20% fetal bovine serum (FBS; Atlanta Biologicals, Lawrenceville, GA), 100 μM 2-mercaptoethanol (Sigma-Aldrich, http://www.sigmaaldrich.com), 100 U/ml penicillin and 100 μg/ml streptomycin (Atlanta Biologicals, Lawrenceville, GA) to make a single-cell suspension. It was then cultured in either tissue culture flasks or plates at 37 °C with 5% CO_2_.

For BMSCs, two milliliters of bone marrow was aspirated with an 18-gauge needle that was fastened to a 5-ml syringe containing 0.2 ml of heparin (1,000 units/ml). The aspirates were washed twice with phosphate-buffered saline (PBS) and centrifuged at 1,500 rpm for 5 min. The supernatant was discarded. The cells were re-suspended in growth medium and incubated in either tissue culture flasks or plates at 37 °C in a humidified 5% CO_2_–95% air atmosphere. The cell colonies were stained with methyl violet (Sigma-Aldrich, http://www.sigmaaldrich.com). Colony numbers were counted manually. Each colony was trypsinized locally under microscopic visualization in order to detach stem cell colonies and detached cells were collected.

The total cell numbers from each colony were counted using a hemocytometer and transferred to T25 flasks for further culture.

### Expression of stem cell markers of MMSCs and BMSCs

Immunocytochemistry was used to assay for expression of the following stem cell markers: nucleostemin, Nanog, SSEA-4, CD34, CD44, CD90 and STRO-1. To perform immunostaining, MMSCs and BMSCs at passage 1 were seeded in 12-well plates at the density of 3 × 10^4^/well and cultured with growth medium for three days.

After removing the medium, the cells were washed with PBS once. MMSCs and BMSCs were fixed with 4% paraformaldehyde in PBS for 30 min at room temperature and treated with 0.1% Triton X-100 for 30 min for Nanog and nucleostemin staining. After washing the cells with PBS, either mouse anti-Nanog (1:350, Santa Cruz Biotechnology, Inc., cat. # SC-33759, Santa Cruz, CA) or goat anti-nucleostemin (1:400, Neuromics, Cat. # GT15050, Edina, MN) was applied to the cells. In order to stain for SSEA-4 and strol-1, fixed cells were incubated either with mouse anti- SSEA-4 antibody (1:500, Invitrogen, Cat. # 414000, Frederick, MD), or mouse anti-strol-1 antibody (1:400, Cat. #398401, Invitrogen, Carlsbas, CA). After 2 hours reaction at room temperature, the cells were washed with PBS for three times, and either Cy-3-conjugated goat anti-mouse IgG antibody (1:500 for Nanog, SSEA-4 and strol-1, Millipore, Cat. # AP124C, Billerica MA) or Cy3-conjugated donkey anti-goat IgG antibody (1:500 for nucleostemin, Millipore, Cat. # AP180C, Billerica, MA) was applied for 1 h at room temperature.

In addition, stem cell surface markers CD34, CD44 and CD90 were stained in parallel by immunocytochemistry. Briefly, fixed cells were incubated with fluorescein isothiocyanate (FITC)-conjugated mouse anti-CD34, or FITC-conjugated mouse anti-CD44, or phycoerythrin (PE)-conjugated mouse anti-CD90 antibodies (1:400) at room temperature for 1 hour. Antibodies were purchased from BD Pharmingen (BD Biosciences; http://bdbiosciences.com), Stem cell Technologies (Vancouver, BC) and Santa Cruz Biotechnology (Santa Cruz, CA), respectively. Fluorescent images of the stained cells were taken by a CCD camera on an inverted fluorescent microscope (Nikon eclipse, TE2000-U) using SPOT™ imaging software (Diagnostic Instruments Inc., Sterling Heights, MI). A total of 36 views from 3 wells of a 12-well plate were randomly chosen for each stem cell marker and the number of positively stained cells was manually counted. The percentage of each stem cell marker expression was determined by dividing the number of positively stained cells by the total number of cells stained by the nuclear staining reagent Hoechst fluorochrome 33342 (1 μg/ml; Sigma, St. Louis, MO).

### Multi-differentiation potential of MMSCs and BMSCs in Vitro

Multi-differentiation potential of MMSCs and BMSCs *in vitro* were tested for adipogenesis, chondrogenesis, and osteogenesis. Both types of cells at passage 2 were seeded either on plastic surfaces in 6-well plates at a density of 2.4 × 10^5^ cells/well or in 24-well plates at a density of 6 × 10^4^ cells/well in basic growth medium consisting of low glucose DMEM, 10% heat inactivated FBS, 100U/ml penicillin, and 100 μg/ml streptomycin. To test adipogenic potential, cells were cultured in adipogenic induction medium (Millipore, Billerica, MA) consisting of basic growth medium added with dexamethasone (1 μM), insulin (10 μg/ml), indomethacin (100 μM), and isobutylmethylxanthine (0.5 mM). As a test of chondrogenic potential, two kinds of MSCs were cultured in basic growth medium supplemented with prolin (40 μg/ml), dexamethasone (39 ng/ml), TGF-β3 (10 ng/ml), ascorbate 2-phosphate (50 μg/ml), sodium pyruvate (100 μg/ml), and insulin transferrin-selenious acid mix (50 mg/ml) (BD Bioscience, Bedford, MA). Finally, the osteogenic potential of MMSCs and BMSCs was tested by culturing them in osteogenic induction medium (Millipore, Billerica, MA) consisting of basic growth medium supplemented with dexamethasone (0.1 μM), ascorbic 2-phosphate (0.2 mM), and glycerol 2-phosphate (10 mM).

### Histochemical analysis

After culturing for 21 days, MMSCs and BMSCs grown in 24-well with various differentiation media were stained using Oil Red O for adipogenesis, Safranin O for chondrogenesis, or Alizarin Red S for osteogenesis, respectively. The stained samples were examined using an inverted microscope as we depicted above. The ratio of positive staining was calculated by dividing the stained area by the view area. The values of all views from three duplicate wells were averaged to obtain the percentage of positive staining, which represents the extent of cell differentiation in the respective induction medium.

### Quantitative real-time PCR (qRT-PCR)

The specific gene expression of differentiated MMSCs and BMSCs were determined using qRT-PCR. Total RNA was extracted using a RNasy Mini-Kit with an on-column DNase I digest (Qiagen). First-strand cDNA was synthesized in a 20 μl reaction of 1 μg total RNA through reverse transcription with Super-Script II (Invitrogen). The conditions for the cDNA synthesis were: 65 °C for 5 min and cooling for 1 min at 4 °C, then 42 °C for 50 min, and finally 72 °C for 15 min. The qRTPCR was carried out using QIAGEN QuantiTect SYBR Green PCR Kit (Qiagen) [11]. In a 25 μl PCR reaction mixture, 2 μl cDNA (total 100 ng RNA) were amplified in a Chromo 4 Detector (MJ Research). Rabbit-specific primers for differentiated cells were used for collagen type II, peroxisome proliferators-activated receptor γ (PPARγ), Sox9, osteocalcin, and Runx2. Glyceraldehyde-3-phosphate dehydrogenase (GAPDH) was used as an internal control. The forward and reverse primer sequences and the resultant products were designed according to published methods, and are listed in Additional file 1: Table S1 [12-15]. All primers were synthesized by Invitrogen (Carlsbad, CA). The relative gene expression levels were calculated from 2^-∆CT^, where ∆CT was determined by the formula: ∆CT = (CTtarget -CT_GAPDH_)_differentiation_-(CT_target_ -CT_GAPDH_)_control_. In the formula, CT_target_ and CT_GAPDH_ are the cycle thresholds of target gene and GAPDH gene, respectively, for each RNA sample. The standard deviation (SD) of the ∆CT was determined from at least three parallel tests.

### Western blot

Two kinds of MSCs were seeded in 6-well plates at a density of 2.4 × 10^5^ per well and cultured with adipogenic, osteogenic and chondrogenic induction media for 21 days, respectively. Then MMSCs and BMSCs were lysed using a mammalian protein extraction reagent cocktail (Pierce, Rockford, Illinois) containing 1.5% protease inhibitors (Sigma-Aldrich). After centrifugation at 12,000 rpm for 10 minutes, the protein concentrations of the supernatants were determined using a BCA Protein Assay kit (Pierce). Equal amounts of total protein were run on 12% SDS polyacrylamide gels (Bio-Rad) at a constant voltage of 100 V for 60 minutes. Proteins were blotted to a nitrocellulose membrane using a Semi-Dry transfer module (Bio-Rad) at 200 mA for 90 minutes. The membrane was blocked in a 5% dry milk/TBS-Tween 20 solution for 1 hour at room temperature and then probed for 5 hours with a mouse monoclonal anti-adiponectin antibody (Millipore; Cat #MAB3604) at a dilution of 1:1000; mouse monoclonal anti-osteocalcin (Abcam; Cat #ab13418) at a dilution of 1:1000 for 5 hours; and mouse anti-collagen II (Millipore; Cat # MAB8887) at a dilution of 1:500 in a 1% dry milk/PBS-Tween 20 solution. Incubation with the primary antibody was followed by a horseradish peroxidase-conjugated goat anti-mouse antibody (Millipore; Cat #12-349) at a dilution of 1:2000 in a 1% dry milk/PBS solution. The targeted protein bands were detected using an ECL (enhanced luminol-based chemiluminescence) detection kit (Amersham Biosciences, Piscataway, New Jersey), followed by exposure of the membrane to X-ray film. Membranes were also re-probed for mouse anti-glyceraldehyde-3-phosphate dehydrogenase (GAPDH, EMD Millipore, Merck KGaA, Darmstadt, Germany; Cat # MAB374) to verify equal protein loading in the gels. The band intensity was quantified by image J software (http://rsb.info.nih.gov/nih-image/).

### Histochemical analysis of wounded meniscus sections

The menisci were obtained aseptically from female New Zealand white rabbits within 12 hours of death. A wound with 1 mm diameter was created in the center of each meniscus by a biopsy punch (Miltex, Inc., Cat. #REF33-31AA, York, PA). Either BMSCs or MMSCs at passage 2 were seeded in these defects, culturing with 10% FBS-DMEM for 6 weeks. The culture medium was changed every 3 days.

Six weeks later the wounded meniscus samples were harvested and placed in pre-labeled base molds filled with frozen section medium (Neg50; Richard-Allan Scientific; Kalamazoo, MI). The tissue samples in base mold filled were then quickly immersed in liquid nitrogen cold-2-methylbutane and allowed to solidify completely. Then the tissue blocks were placed on dry ice and subsequently stored in the −80 °C freezer until histological analysis was carried out.

The tissue block was cut into 10 μm thick sections and placed on glass slides, and then these glass slides were left over night at the room temperature to dry. The sections were rinsed 3 times with PBS and fixed with 4% paraformaldehyde for 30 min, then washed with PBS for another 3 times. The chondrogenesis differentiation of BMSCs and MMSCs on wounded rabbit meniscus was tested by histochemical staining. The sections were stained with alcian blue, toluidine blue, safranin O and fast-green. All images were taken using a CCD camera as we depicted above.

### In vivo implantation experiments

Four female nude rats (10 weeks old; 200 – 250 g) were used to test the differentiation of two kinds of MSCs *in vivo*. Rats were housed individually on a 12 h : 12 h light–dark cycle and were cared for in accordance with the Guide to the Care and Use of Experimental Animals. The stem cells at passage 2 with the density of 6 × 10^4^ cells/0.1 ml were mixed with 0.5 ml of Matrigel (BD 201 Biosciences, Cat. # 354234, Bedford, MA) in a 24-well plate and overnight cultured with DMEM-10% FBS at 37 °C and 5% CO_2_. In the next day, the medium was removed from each well and the Matrigel with cells were used for implantation into nude rat skin.

The nude rats were placed under general anesthesia using ketamine hydrochloride (75 mg/kg body weight) and xylazine hydrochloride (5 mg/kg body weight), administrated by intramuscular injection. Wounds (1 cm diameter/each wound) were created on the back of each rat, and three pieces of cell-Matrigel were placed in three distinct wounds on each side of the rat’s back. The approximate distance between two wound sites was 1.5 cm, with a total of six cell-Matrigel composites implanted into each rat. Each group had two rats and a total of four rats were used for two groups. At 3 weeks after implantation, tissue samples were harvested and frozen according to the measure we described above.

The tissue block was cut into 10 μm thick sections, which were then placed on glass slides and allowed to dry overnight at room temperature. The multi-differentiation potentials of two kinds of MSCs *in vivo* were tested by immunostaining on tissue sections of nude rats after implantation. The tissue sections were fixed with 4% paraformaldehyde for 15 min and reacted with mouse anti-adiponectin (1:300, Millipore; Cat. #MAB3604; Temecula, CA), mouse anti-osteocalcin (1:200, Abcam, Cat #13418; Cambridge, MA), mouse anti-collagen type I (1:100, Millipore, Cat. #MAB1340; Temecula, CA), and mouse anti-collagen type II (1:100, Millipore, Cat. #MAB1330, Temecula, CA) at room temperature for 2 hours. FITC-conjugated goat anti-mouse IgM (1:500, Santa Cruz Biotechnology, Cat. #sc-2082, Santa Cruz, CA) was used as the secondary antibody to detect adiponectin and Cy-3 conjugated goat anti-mouse IgG (1:500, Jackson ImmunoResearch Laboratories, Inc., Cat. #115-165-146, West Grove, PA) was used as the secondary antibody to detect collagen type I, collagen type II and osteocalcin at room temperature for 2 hrs. The tissue sections were also treated with Hoechst 33342 (Sigma, Cat. #B2261, St. Louis, MO) to stain nuclei.

### Statistical analysis

Data is presented as mean plus and minus standard deviation (SD). At least three replicates for each experimental condition were performed, and the presented results are representative of these replications. One-way analysis of variance (ANOVA), followed by either Fisher’s predicted least-square difference (PLSD) for multiple comparisons or two tailed student t-test wherever applicable, were used for statistical analysis. Differences between two groups were considered significant when the p-value was less than 0.05.

## Results

### Colony formation

**Figure 1:**
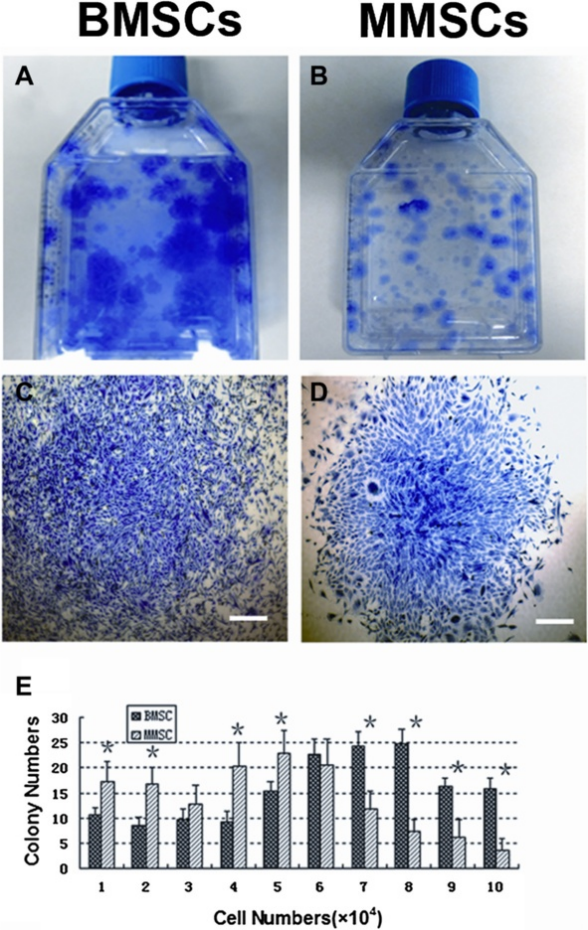
**The colony formation of bone marrow**-**derived stem cells** **(BMSCs)** **and meniscus-** **derived stem cells** **(MMSCs).** **A**. Colonies of BMSCs. **B**. Colonies of MMSCs. **C**. A sample colony of BMSCs. **D**. A sample colony of MMSCs. **E**. Quantitative analysis of colonies formed by BMSCs and MMSCs. The colonies were detected by staining with Methyl violet at 15 days primary culture. Cell numbers were counted after trypsinized from each colony respectively. Colony number of MMSCs was significantly different from that of BMSCs (*p < 0.05). (Bars: 50 μm).

To characterize whether meniscus-derived cells are clonogenic, we isolated and cultured single suspension from rabbit meniscus and compared with bone marrow-derived cells in five T25 flasks. During the initial three days in culture, these cells began to attach onto the plastic surface and remained quiescent for approximate five days. After 8–10 days of culture, the first colony was observed in each flask. Then large quantities of cells started rapidly dividing to form considerable colonies at 10–15 days. Methyl violet staining was used to discover the colony (Figure 1 A, B). However, the number and size of cell colonies from BMSCs and MMSCs were markedly different: colonies formed by MMSCs were fewer in number and smaller in size than those of BMSCs. Our data showed that only 51.8% of colonies consisted of 50,000 cells or more in MMSCs whereas 75.8% in BMSCs (Figure 1E).

### Immunocytochemistry analysis of MMSCs and BMSCs

**Figure 2:**
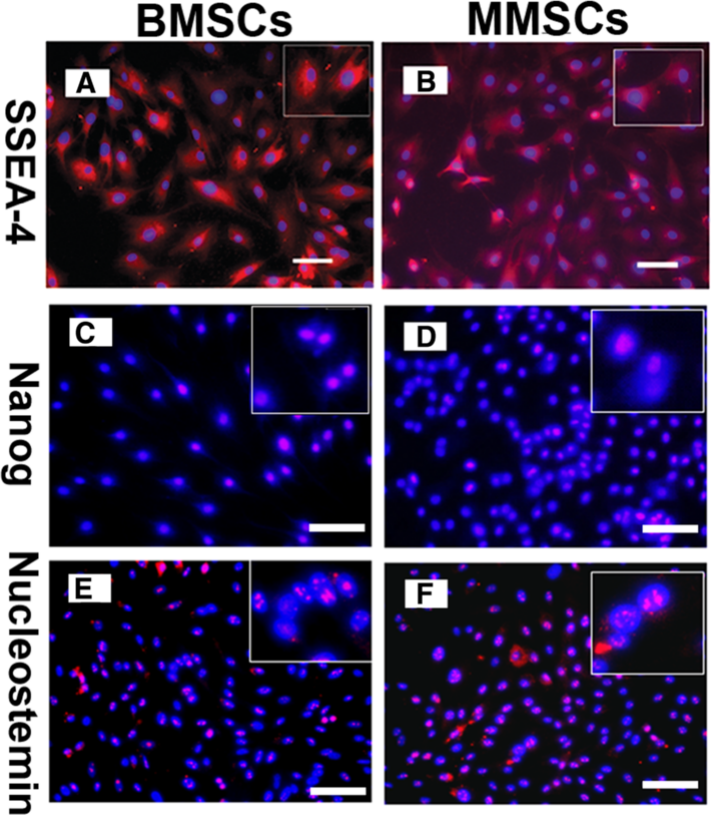
**Expression of stem cell markers for BMSCs and MMSCs.** Both types of MSCs exhibited high expression of stem cell markers, SSEA-4 **(A, B)**, Nanog **(C, D)**, and nucleostemin **(E, F)**, respectively. Insets show enlarged view of positive staining with three stem cell markers. There was no great difference in the expression of these three recognized stem cell markers. (Magnification of microscopy: 20×) (Bar: 50 μm).

**Figure 3:**
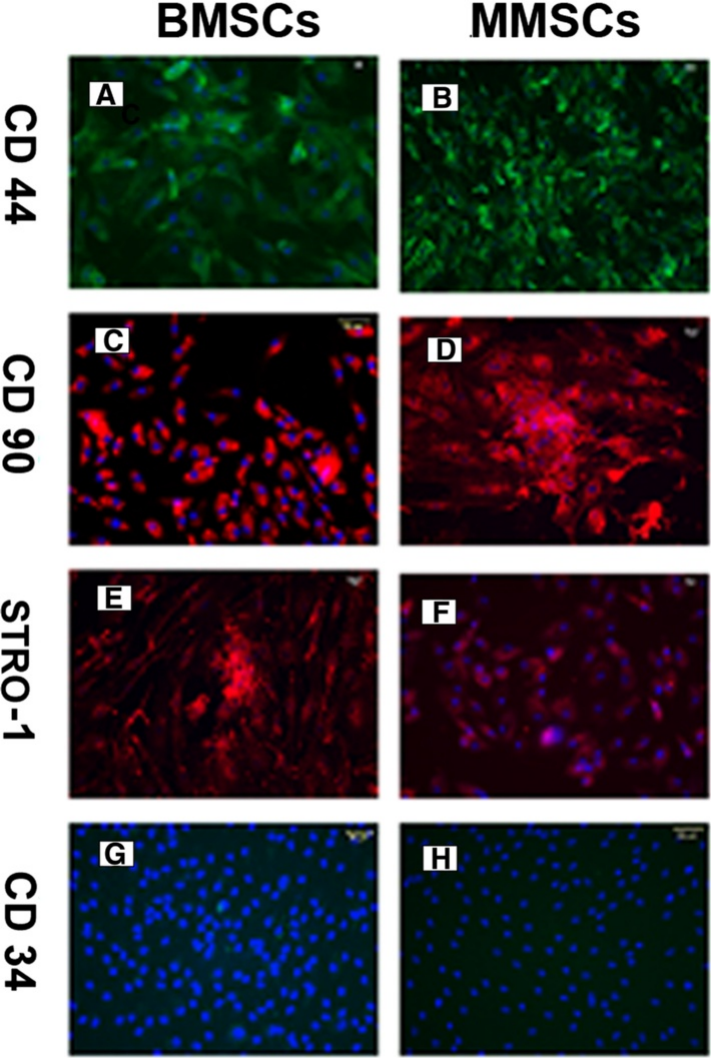
**Expression of MSC markers for BMSCs and MMSCs.** All three MSC markers, i.e. CD 44, CD 90 and Strol-1, were strong expressed in BMSCs **(A, C and E)** and MMSCs **(B, D and F)**. Additionally, CD 34, a hematopoietic cell marker was negative stained in MMSCs **(H)** and lower 3% positively stained in BMSCs **(G)**. (Magnification of microscopy: 20×) (Bar: 50 μm)

**Figure 4:**
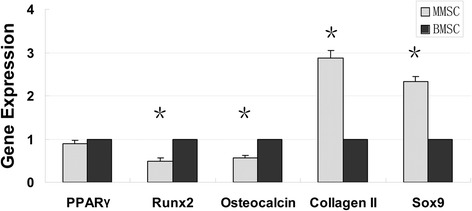
**The qRT**-**PCR analysis of adipogenic**, **osteogenic and chondrogenic marker genes.** Much higher expression of chondrogenic gene markers including collagen type II and Sox9 were observed in MMSCs group (**P < 0.01). However, BMSCs expressed much higher level of osteogenic gene markers such as osteocalcin and Runx-2 (**P < 0.01) No great difference was found on expression of adipogenic gene marker. Note that the gene expression levels were normalized to GAPDH, and obtained from at least three independent experiments.

To confirm whether MMSCs possess the established properties of stem cells, we examined the stem cell markers through immunochemistry staining. Over 80% positively stained cells were found for SSEA-4, Nanog and nucleostemin in both MMSCs and BMSCs groups (Figure 2). Furthermore, about 86% of MMSCs and 82% of BMSCs stained positively for STRO-1 (Figure 3E, F). In addition, more than 85% of cells from these two groups were found to be positively stained by CD44 and CD90 (Figure 3A-D), while lower 3% of BMSCs were positively stained by CD34 and only few MMSCs were found positive by CD34 (Figure 4G, H).

### Multipluripotent evaluation of MMSC and BMSCs in vitro

We next examined whether MMSCs possessed the capacity of differentiating into various lineages compared to that of BMSCs. The multidifferentiation potential of MMSCs and BMSCs towards adipogenesis, osteogenesis and chondrogenesis were determined through histological staining, qRT-PCR and western blot.

**Figure 5:**
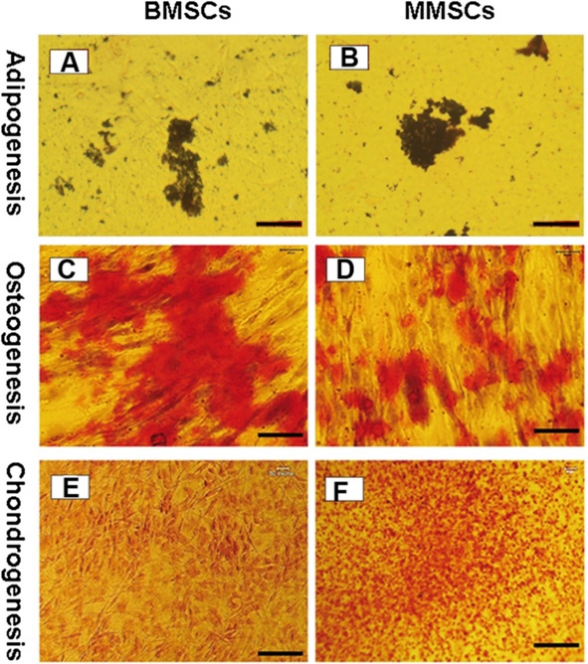
**Histochemical staining of differentiated cells and semi**-**quantification of the extent of cell differentiation.** Both BMSCs and MMSCs were able to differentiate into adipocytes **(A, B)**, osteocytes **(C, D),** and chondrocytes **(E, F)**, as shown by the accumulation of lipid droplets, proteoglycans and calcium deposits on cell surfaces. However, higher extent of osteogenic differentiation in BMSCs was evidenced by the most positive staining areas through Alizarin Red S assay. Conversely, much higher potential of chondrogenic differentiation in MMSCs was verified by Safranin O staining. Note that each experiment was repeated three times using five different donors. (P < 0.05) (Magnification of microscopy: 20×) (Bar: 50 μm).

After 21 days in adipogenic medium, both MMSCs and BMSCs exhibited numerous lipid droplets, an indicator of adipogenesis which can be detected by Oil Red O staining. Semiquantitative evaluation, by calculating stained area, showed that about 24% of MMSCs and 28% of BMSCs were differentiated into adipocytes, respectively (Figure 5A, B). Similar to this result, on the expression level of PPARγ, a gene marker of adipogenic lineage, MMSCs was akin to BMSCs (Figure 4).

When cultured in osteogenic medium, BMSCs spontaneously began to form large aggregates in the plastic plate at 15 days. In regard to this, we collected both types of stem cells at 14 days, and detected the expression of calcium using Alizarin Red S assay. More than 44% of BMSCs were stained positively compared to 32% of MMSCs (Figure 5C, D). Similarly, qRT-PCR analysis showed that the expression of the osteogenic markers osteocalcin and Runx-2 was all significantly higher in BMSCs than that in MMSCs (Figure 4).

In regard to chondrogenesis, similarly to the previously described observation related to the behavior of BMSCs during osteogenic induction, MMSCs began to form aggregates at 13 days, and then recruited cells continually from circumference to generate a consolidated spherical tissue ultimately. Consequently, the two different cell populations were collected at 12 days and examined expression of glycosaminoglycans (GAG)-rich matrix using Safranin O staining. About 46% positive staining in MMSCs appeared while only 32% in BMSCs (Figure 5E, F).

Equally, MMSCs expressed higher levels of collagen type II (2.8 folds) and Sox9 (2.3 folds) which are two gene markers for chondrogenesis than BMSCs (Figure 4).

**Figure 6:**
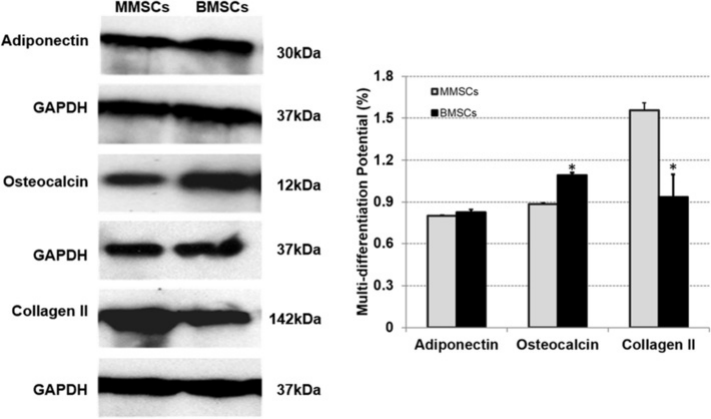
**Representative western blots of differentiated BMSCs and MMSCs.** Collagen type II, the most important indicator for chondrogenesis, was expressed much higher in MMSCs than that in BMSCs. Meanwhile, BMSCs displayed higher expression of osteocalcin, a typical production of osteogenesis. Note that our data were normalized to GAPDH, and obtained from at least three independent experiments. (P < 0.05).

Western blotting was performed to quantify the level of specific protein expression in the two groups of stem cells. Adiponectin was expressed in both MMSCs and BMSCs following 21 days culture in adipogenic induction medium and there was no significant difference between two groups. As for osteocalcin, a well-known marker of osteogenesis, it was markedly up-regulated in BMSCs compared with MMSCs. Conversely, after cultured in chondrogenic medium, MMSCs expressed significantly higher levels of collagen type II which is one of the most important indicators on chondrogenesis than BMSCs (Figure 6).

### The effect of rabbit MMSCs and BMSCs on wounded meniscus healing

**Figure 7:**
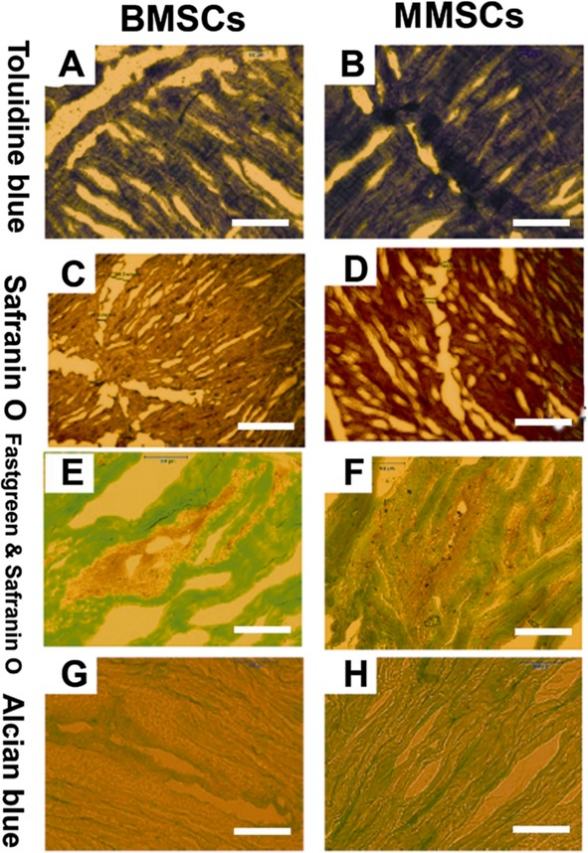
**Histochemical staining on wounded meniscus cultured with BMSCs or MMSCs for 6 weeks.** The meniscus was cut into 10 μm section and stained by toluidine blue **(A, B),** safranin O **(C, D),** fast green and safranin O **(E, F),** alcian blue **(G, H)**. All imaging showed that more cartilage-related proteins were detected in the meniscus treated with MMSCs than BMSCs. (P < 0.05) (Magnification of microscopy: 20×) (Bar: 50 μm).

After 6 weeks culture, more than 90% of the wound area in rabbit meniscus was healed by MMSCs treatment; instead, by means of BMSCs treatment, 80% only was healed. Furthermore, more cartilage-related proteins were formed in the meniscus treated by MMSCs than that treated by BMSCs, as observed with staining using toluidine blue (dark brown in Figure 7B), safranin O (larger red area in Figure 7D), fast green and safranin O (larger red area in Figure 7F) and alcian blue (extensive green area in Figure 7H).

### Immunostaining assay of cell- Matrigel composite

**Figure 8:**
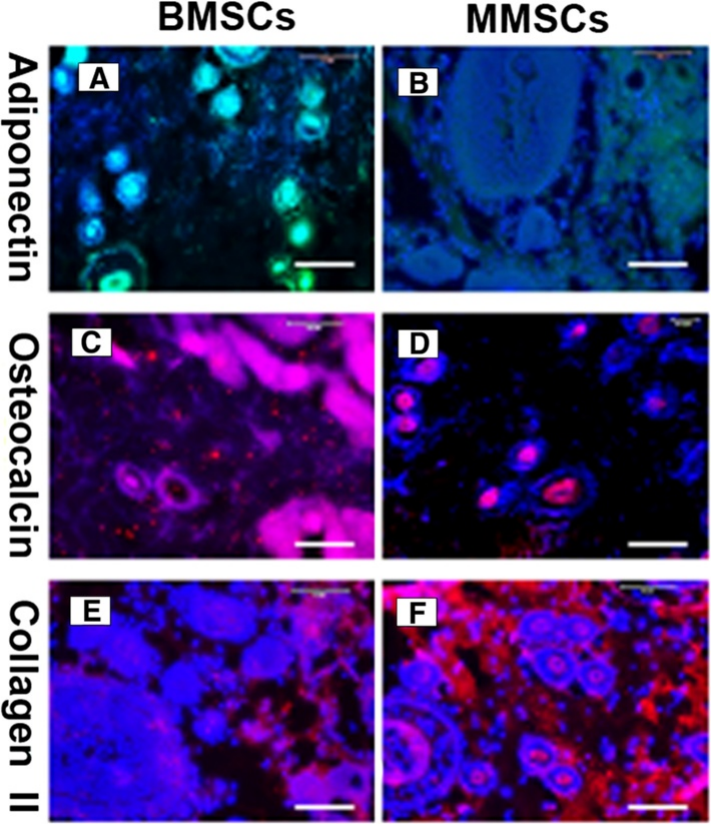
**Immunocytochemistry staining on complexes implanted Matrigel with BMSCs or MMSCs into the back of nude rats for 3 weeks.** The tissue sections were stained with anti-adiponectin **(A, B)**, anti-osteocalcin **(C, D)**, anti-collagen type II **(E, F)**, respectively. It is seen that two kinds of stem cells have multi-differentiation potential *in vivo*. The implantation of BMSCs resulted in more bone-like tissue formation (C, pink) than MMSCs **(D)**. More cartilage-like tissues were observed in MMSCs group, as shown by immunostaining for collagen type II (**F**, red). (P < 0.05) (Magnification of microscopy: 20×) (Bar: 50 μm).

The differentiation capability *in vivo* was examined by implanting these two kinds of MSCs into nude rats. The results obtained from experiments showed that when MMSCs and BMSCs were subcutaneously implanted with Matrigel into the back of nude rats, in samples implanted with Matrigel-MSCs, greater positively for osteocalcin was observed in the samples made of Matrigel-BMSCs (Figure 8C) compared to those with Matrigel-MMSCs (Figure 8D), while the samples with Matrigel-MMSCs expressed much more collagen type II protein (Figure 8F) than those with Matrigel-BMSCs (Figure 8E). As for expression of adipogenesis, no significant difference were found between BMSCs and MMSCs.

## Discussion

The aim of the study was to investigate if MSCs may reside in rabbit menisci and if there is any difference between these meniscus-derived MSCs and bone marrow-derived MSCs. Towards this aim, MMSCs and BMSCs were isolated from rabbit menisci and bone marrow, and their differentiation potential, stem cell marker expression, colony formation were examined. Although the basic cellular architecture of rabbit meniscus has been established previously, there is no report on stem cells isolated rabbit menisci [16,17]. Furthermore, mesenchymal stem cells were initially isolated from bone marrow, it has already gained acceptance that they reside in different adult connective tissues such as synovium, periosteum, adipose tissue, and muscle [18-21].

Actually, a recent study demonstrated that a unique cell subpopulation with the typical characteristics of mesenchymal stem cells resided within rabbit meniscus. These may effectively protect the joint surface and maintained joint space width in an experimental OA model [22]. However, whether these meniscus-derived cells are stem cells and what are the differential properties between these meniscus-derived cells and BMSCs remain largely undefined.

Clonogenicity is an important trait of adult stem cells including neural, hematopoietic, tendon, and epidermal stem cells, as well as for embryonic stem cells [19,23-26]. High frequency of colony formation in MMSCs and BMSCs denotes that large amount of mesenchymal stem cells or progenitor cells reside in the meniscus as well as in the bone marrow.

Our results showed that both populations expressed high level of characteristic stem cell markers including SSEA-4, Nanog and nucleostemin. However, there is no significant difference found in immunostaining results between BMSCs and MMSCs (P > 0.05). These findings indicated that the stem cells isolated from rabbit meniscus have similar properties to those of BMSCs.

SSEA-4, a stage-specific embryonic antigen previously thought to mark specifically human embryonic stem cells and very early cleavage to blastocyst stage embryos, also marks an adult mesenchymal stem cell population [27]. Nanog is only expressed in embryonic stem cells (ESCs) and is thought to be a key factor in maintaining pluripotency [28]. Nucleostemin is a kind of nucleolar protein which is abundantly expressed while the cells are proliferating in an early, multipotential state, but it abruptly and almost entirely disappears at the beginning of the differentiation stage. It is also indicated that this type of protein plays a role in maintaining stem cell self-renewal and regulating the proliferation of stem cells [29,30].

Stro-1 is a surface antigen found on bone marrow mononuclear cells capable of differentiating into osteogenic, chondrogenic and adipogenic lines [31]. It is well known that MSCs express CD44 and CD90, but lack expression of CD34 [32]. Our results agree with the criteria to define MSC. Taken together, the substrate-dependent expression of the above stem cell markers protein reveals that MMSCs still remain undifferentiated stem cells and preserve self-renewal capability similarly to BMSCs.

According to the suggestion by the Mesenchymal and Tissue Stem Cell Committee of the International Society for Cellular Therapy, another basic criteria required for MSC is that they must possess the potential to differentiate into osteoblasts, adipocytes and chondroblasts [32]. Whether *in vitro* or *in vivo*, our findings display that, similar to BMSCs, MMSCs undoubtedly exhibit their multi-potent properties. Furthermore, MMSCs showed a promising chondrogenic differentiation potential compared to BMSCs. Collectively, these data suggest that both BMSCs and MMSCs share common features of MSC populations but display some peculiarities related to their unique differentiating potential. There is ample evidence that even partial meniscectomy greatly changes knee biomechanics and increases the contact pressure on the articular cartilage [33]. The overriding problem with a meniscal tear is the limited capacity that the meniscus has to heal itself effectively, especially in the avascular zone [34]. However, the inability of surgeons to repair the damaged meniscus--both anatomically and functionally, continues to present challenges [35].

Regarding meniscus repair, previous studies have reported that transplanted MSCs from green fluorescent protein transgenic (GFP) rats into meniscal defect could survive and contribute to synthesis of extracellular matrix [36]. Recently, Pabbruwe et al. described beneficial effects on meniscal regeneration by using the stem cell/collagen-scaffold implant [7]. Zellner et al. demonstrated that the repair of punch defects in the avascular zone of the meniscus was achieved with the combination of biodegradable composite matrices and non-precultured BMSCs [8].

Since MSCs can be easily isolated from bone marrow and other sources, it was originally thought that after the delivery of culture-expanded MSCs to the injured host, they would migrate to the site of injury and directly differentiate into the cells of an appropriate phenotype and function, thus contributing to the repair of the injured tissue [37].

However, many studies have demonstrated that MSCs could mediate robust tissue repair, but exhibited low or/and transient engraftment into the injured tissue [38]. It has been reported that BMSCs express cardic-specific markers, retain the stromal phenotype, but they do not become functional cardiomycytes *in vitro* [39]. Although several studies have shown that MSCs may acquire differentiated phenotype, they lack functional activity of specialized cells [40,41]. Moreover, BMSCs suffer from a main drawback: they appear to have a high propensity for cartilage hypertrophy and bone formation, and therefore may not be ideal chondro-progenitors for the repair of meniscus [9,10,20,42-44].

Superior chondrogenic differentiation potential of MMSCs makes this cell population be a reservoir of stem cells which offer a promising option treating damaged meniscus through cell therapy. MSCs derived from various mesenchymal tissues contain common features, but an increasing number of reports describe distinguishing properties dependent on their origin [45,46]. MMSCs may have an important role in repairing meniscus tear in the future. First, homing trait of stem cells determines that MMSCs are more prone to migrate to the meniscus defect than any other stem cells [47]. Second, as meniscus-specific stem cells, MMSCs can by default differentiate into fibroblast-like cells or chondrocyte-like cells naturally. Our results may suggest that it is easier for MMSCs to control the process of differentiation into chondrogenic cells than any other kinds of MSCs. Finally, compared to other MSCs populations, MMSCs may adapt to the niche of the meniscus during the long-term repairing process due to their meniscus originated [42]. Our study showed that (1) meniscus tissues contain cells with stem-cell character, (2) these meniscus-derived cells possess excellent chondrogenic differentiation potential compared to BMSCs, and (3) this specific traits distinct from BMSCs may promote the regeneration of wounded meniscus.

A few limitations in the present study still exist. First, rabbit meniscus is different from human on the main structural features, including cellular distribution, vascularity, and collagen structure [47]. Hence, in order to accurately demonstrate the efficacy of MMSCs on human meniscus repair, larger animal models are required for further studies. Second, cells used in this study were isolated from the whole meniscus of young rabbits (8–10 weeks old), however, two distinct regions of meniscus have been distinguished based on different vascularization states. Whether there are some differences between two MMSCs populations harvested from vascular region and avascular region remains indeterminate. More precise isolation of MMSCs from different regions of meniscus and different ages of animals should be adopted in the future. Third, this study didn’t investigat the effect of MMSCs on wounded meniscus healing *in vivo*. In the future study, we will use nanostructured scaffold to deliver MMSCs into the wounded meniscus of large animals and investigated aging effect on MMSCs for meniscus repair and regeneration.

## Conclusions

Our study demonstrated that both BMSCs and MMSCs share common features of MSCs populations in colony formation and multi-differentiation potential. Comparing to BMSCs, MMSCs may serve as an alternative cell therapy in repairing damaged meniscus due to their homing traits and promising potential on chondrogenic differentiation which conduce them to adapt to the niche inside the meniscus. The present results indicated that the stem cells isolated from meniscal may be used for an allogeneic transplantation through scaffold for meniscal repair in human.

## Additional file

Additional file 1: Table S1. Primers used for qRT-PCR analysis. http://www.biomedcentral.com/imedia/1571346029144213/supp1.doc.

## Abbreviations

BMSCs: Bone marrow-derived mesenchymal stem cells
MSCs: Mesenchymal stem cells
MMSCs: Meniscus-derived mesenchymal stem cells
SSEA-4: Stage specific embryonic antigen-4
MSCs --- SMSCs: Synovium-derived
MSCs --- AMSCs: Adipose-derived
PPARγ: Peroxisome proliferators-activated receptor γ
GAPDH: Glyceraldehyde-3-phosphate dehydrogenase.

## References

[1] Cook JL (2005). The current status of treatment for large meniscal defects. Clin Orthop Relat Res.

[2] Higuchi H, Kimura M, Shirakura K, Terauchi M, Takagishi K (2000). Factors affecting long-term results after arthroscopic partial meniscectomy. Clin Orthop Relat Res.

[3] Kozlowski EJ, Barcia AM, Tokish JM (2012). Meniscus repair: the role of accelerated rehabilitation in return to sport. Sports Med Arthrosc.

[4] Sweigart MA, Athanasiou KA (2001). Toward tissue engineering of the knee meniscus. Tissue Eng.

[5] McAndrews PT, Arnoczky SP (1996). Meniscal repair enhancement techniques. Clin Sports Med.

[6] Buma P, Ramrattan NN, van Tienen TG, Veth RP (2004). Tissue engineering of the meniscus. Biomaterials.

[7] Pabbruwe MB, Kafienah W, Tarlton JF, Mistry S, Fox DJ, Hollander AP (2010). Repair of meniscal cartilage white zone tears using a stem cell/collagen-scaffold implant. Biomaterials.

[8] Zellner J, Mueller M, Berner A, Dienstknecht T, Kujat R, Nerlich M (2010). Role of mesenchymal stem cells in tissue engineering of meniscus. J Biomed Mater Res.

[9] De Bari C, Dell'Accio F, Karystinou A, Guillot PV, Fisk NM, Jones EA (2008). A biomarker-based mathematical model to predict bone-forming potency of human synovial and periosteal mesenchymal stem cells. Arthritis Rheum.

[10] Ruiz-Iban MA, Diaz-Heredia J, Garcia-Gomez I, Gonzalez-Lizan F, Elias-Martin E, Abraira V (2011). The effect of the addition of adipose-derived mesenchymal stem cells to a meniscal repair in the avascular zone: an experimental study in rabbits. Arthroscopy.

[11] Claus R, Lacorn M, Welter H, Lekhkota O, Messe N, Wagner A (2007). Expression of 11beta-hydroxysteroid-dehydrogenase 2 in Sertoli cells of boar testes. Mol Cell Endocrinol.

[12] Zhao SP, Dong SZ (2008). Effect of tumor necrosis factor alpha on cholesterol efflux in adipocytes. Clin Chim Acta.

[13] Emans PJ, Spaapen F, Surtel DA, Reilly KM, Cremers A, van Rhijn LW (2007). A novel in vivo model to study endochondral bone formation; HIF-1alpha activation and BMP expression. Bone.

[14] Intawicha P, Ou YW, Lo NW, Zhang SC, Chen YZ, Lin TA (2009). Characterization of embryonic stem cell lines derived from New Zealand white rabbit embryos. Cloning Stem Cells.

[15] Martins A, Pinho ED, Correlo VM, Faria S, Marques AP, Reis RL (2010). Biodegradable nanofibers-reinforced microfibrous composite scaffolds for bone tissue engineering. Tissue Eng Part A.

[16] Melrose J, Smith S, Cake M, Read R, Whitelock J (2005). Comparative spatial and temporal localisation of perlecan, aggrecan and type I, II and IV collagen in the ovine meniscus: an ageing study. Histochem Cell Biol.

[17] Hellio Le Graverand MP, Ou Y, Schield-Yee T, Barclay L, Hart D, Natsume T (2001). The cells of the rabbit meniscus: their arrangement, interrelationship, morphological variations and cytoarchitecture. J Anat.

[18] Parker AM, Katz AJ (2006). Adipose-derived stem cells for the regeneration of damaged tissues. Expert Opin Biol Ther.

[19] Bi Y, Ehirchiou D, Kilts TM, Inkson CA, Embree MC, Sonoyama W (2007). Identification of tendon stem/progenitor cells and the role of the extracellular matrix in their niche. Nat Med.

[20] Yoshimura H, Muneta T, Nimura A, Yokoyama A, Koga H, Sekiya I (2007). Comparison of rat mesenchymal stem cells derived from bone marrow, synovium, periosteum, adipose tissue, and muscle. Cell Tissue Res.

[21] Sakaguchi Y, Sekiya I, Yagishita K, Muneta T (2005). Comparison of human stem cells derived from various mesenchymal tissues: superiority of synovium as a cell source. Arthritis Rheum.

[22] Shen W, Chen J, Zhu T, Yin Z, Chen X, Chen L (2013). Osteoarthritis prevention through meniscal regeneration induced by intra-articular injection of meniscus stem cells. Stem Cells Dev.

[23] Pitman M, Emery B, Binder M, Wang S, Butzkueven H, Kilpatrick TJ (2004). LIF receptor signaling modulates neural stem cell renewal. Mol and Cell Neurosci.

[24] Skobin V, Jelkmann W, Morschakova E, Pavlov AD, Schlenke P (2000). Tumor necrosis factor-alpha and TNF-beta inhibit clonogenicity of mobilized human hematopoietic progenitors. J Interferon Cytokine Res.

[25] Chan RW, Schwab KE, Gargett CE (2004). Clonogenicity of human endometrial epithelial and stromal cells. Biol Reprod.

[26] Li L, Wang BH, Wang S, Moalim-Nour L, Mohib K, Lohnes D (2010). Individual cell movement, asymmetric colony expansion, rho-associated kinase, and E-cadherin impact the clonogenicity of human embryonic stem cells. Biophys J.

[27] Gang EJ, Bosnakovski D, Figueiredo CA, Visser JW, Perlingeiro RC (2007). SSEA-4 identifies mesenchymal stem cells from bone marrow. Blood.

[28] Lin T, Chao C, Saito S, Mazur SJ, Murphy ME, Appella E (2005). p53 induces differentiation of mouse embryonic stem cells by suppressing Nanog expression. Nat Cell Biol.

[29] Bernardi R, Pandolfi PP (2003). The nucleolus: at the stem of immortality. Nat Med.

[30] Normile D (2002). Cell proliferation. Common control for cancer, stem cells. Science.

[31] Dennis JE, Carbillet JP, Caplan AI, Charbord P (2002). The STRO-1+ marrow cell population is multipotential. Cells Tissues Organs.

[32] Dominici M, Le Blanc K, Mueller I, Slaper-Cortenbach I, Marini F, Krause D (2006). Minimal criteria for defining multipotent mesenchymal stromal cells. The International Society for Cellular Therapy position statement. Cytotherapy.

[33] Starke C, Kopf S, Petersen W, Becker R (2009). Meniscal repair. Arthroscopy.

[34] Ibarra C, Koski JA, Warren RF (2000). Tissue engineering meniscus: cells and matrix. Orthop Clin North Am.

[35] Pittenger MF, Mackay AM, Beck SC, Jaiswal RK, Douglas R, Mosca JD (1999). Multilineage potential of adult human mesenchymal stem cells. Science.

[36] Yamasaki T, Deie M, Shinomiya R, Izuta Y, Yasunaga Y, Yanada S (2005). Meniscal regeneration using tissue engineering with a scaffold derived from a rat meniscus and mesenchymal stromal cells derived from rat bone marrow. J Biomed Mater Res.

[37] Barry FP, Murphy JM (2004). Mesenchymal stem cells: clinical applications and biological characterization. Int J Biochem Cell Biol.

[38] Pelttari K, Winter A, Steck E, Goetzke K, Hennig T, Ochs BG (2006). Premature induction of hypertrophy during in vitro chondrogenesis of human mesenchymal stem cells correlates with calcification and vascular invasion after ectopic transplantation in SCID mice. Arthritis Rheum.

[39] Rose RA, Jiang H, Wang X, Helke S, Tsoporis JN, Gong N (2008). Bone marrow-derived mesenchymal stromal cells express cardiac-specific markers, retain the stromal phenotype, and do not become functional cardiomyocytes in vitro. Stem Cells.

[40] Khoo ML, Tao H, Meedeniya AC, Mackay-Sim A, Ma DD (2011). Transplantation of neuronal-primed human bone marrow mesenchymal stem cells in hemiparkinsonian rodents. PLoS One.

[41] Goldstein SA (2002). Tissue engineering: functional assessment and clinical outcome. Ann N Y Acad Sci.

[42] Guilak F (2002). Functional tissue engineering: the role of biomechanics in reparative medicine. Ann N Y Acad Sci.

[43] Hofstetter CP, Schwarz EJ, Hess D, Widenfalk J, El Manira A, Prockop DJ (2002). Marrow stromal cells form guiding strands in the injured spinal cord and promote recovery. Proc Natl Acad Sci U S A.

[44] Sakaguchi Y, Sekiya I, Yagishita K, Ichinose S, Shinomiya K, Muneta T (2004). Suspended cells from trabecular bone by collagenase digestion become virtually identical to mesenchymal stem cells obtained from marrow aspirates. Blood.

[45] Mochizuki T, Muneta T, Sakaguchi Y, Nimura A, Yokoyama A, Koga H (2006). Higher chondrogenic potential of fibrous synovium- and adipose synovium-derived cells compared with subcutaneous fat-derived cells: distinguishing properties of mesenchymal stem cells in humans. Arthritis Rheum.

[46] Caplan AI, Dennis JE (2006). Mesenchymal stem cells as trophic mediators. J Cell Biochem.

[47] Chevrier A, Nelea M, Hurtig MB, Hoemann CD, Buschmann MD (2009). Meniscus structure in human, sheep, and rabbit for animal models of meniscus repair. J Orthop Res.

