# Supplementary material for: Mesenchymal stem cells in rabbit meniscus and bone marrow exhibit a similar feature but a heterogeneous multi-differentiation potential: superiority of meniscus as a cell source for meniscus repair
Source: BMC Musculoskelet Disord. 2015 Mar 21;16:65. doi: 10.1186/s12891-015-0511-8 (PMC4373281; doi:10.1186/s12891-015-0511-8)
Supplement: Additional file 1: Table S1. — Primers used for qRT-PCR analysis. http://www.biomedcentral.com/imedia/1571346029144213/supp1.doc. [file 12891_2015_511_MOESM1_ESM.doc]

| **Table 1. Primers used for qRT-PCR analysis** | | |  |  |
| --- | --- | --- | --- | --- |
| **Gene** | **Size (bp)** | **Primers** | **Type** | **Tm** |
| **PPARγ** | 200 | 5’-TGG GGA TGT CTC ATA ATG CCA-3’ | Forward | 59℃ |
|  |  | 5’-TTC CTG TCA AGA TCG CCC TCG-3’ | Reverse |  |
| **Collagen II** | 84 | 5’-TGG GTG TTC TAT TTA TTT ATT GTC TTC CT-3’ | Forward | 63℃ |
|  |  | 5’-GCG TTG GAC TCA CAC CAG TTA GT-3’ | Reverse |  |
| **Sox9** | 79 | 5’-AGT ACC CGC ACC TGC ACA AC-3’ | Forward | 59℃ |
|  |  | 5’-CGC TTC TCG CTC TCG TTC AG-3’ | Reverse |  |
| **Runx2** | 70 | 5’-TGA TGA CAC TGC CAC CTC TGA-3’ | Forward | 58℃ |
|  |  | 5’-GCA CCT GCC TGG CTC TTC T-3’ | Reverse |  |
| **GAPDH** | 107 | 5’-ACT TTG TGA AGC TCA TTT CCT GGT A-3’ | Forward | 63℃ |
|  |  | 5’-GTG GTT TGA GGG CTC TTA CTC CTT-3’ | Reverse |  |
| **Osteocalcin** | 70 | 5’-GAAGCCCAGCGGTGCA-3’ | Forward | 59℃ |
|  |  | 5’-CACTACCTCGCTGCCCTCC-3’ | Reverse |  |
